# From Healing to Regeneration: A Comprehensive Review of the Efficacy of Platelet-Rich Fibrin in Periodontal Plastic Surgery Procedures

**DOI:** 10.7759/cureus.69287

**Published:** 2024-09-12

**Authors:** Amal G Jamjoom

**Affiliations:** 1 Periodontology Department, Faculty of Dentistry, King Abdulaziz University, Jeddah, SAU

**Keywords:** periodontal regeneration, wound healing, root coverage, tissue regeneration, periodontal plastic surgery, platelet-rich fibrin

## Abstract

This review examines platelet-rich fibrin (PRF) efficacy in periodontal plastic surgery, highlighting its crucial role in promoting periodontal regeneration and healing. Various forms of PRF are discussed, like leukocyte- and platelet-rich fibrin, advanced PRF, and injectable PRF, in addition to their application in different periodontal procedures such as root coverage and increasing the width of keratinized tissue surrounding the teeth. This review emphasizes the biological benefits of PRF, such as faster wound healing, reduced postsurgical pain, and better management of bleeding. The presence of growth factors, cytokines, and leukocytes in PRF significantly aids in promoting tissue regeneration, thereby improving the clinical outcomes of periodontal therapy. This review also provides recommendations for further research using standardized PRF protocols to optimize the benefits of PRF in clinical practice.

## Introduction and background

The term "mucogingival" typically refers to the mucogingival junction and related anatomical structures, such as the gingiva, alveolar mucosa, frenula, muscular attachments, vestibular fornices, and the floor of the mouth [1]. Mucogingival surgery, or periodontal plastic surgery, refers to surgical interventions to rectify or remove deformities of the gingiva or alveolar mucosa caused by anatomical issues, developmental irregularities, or injuries [2]. The 1996 World Workshop in Periodontics' Consensus Report on Mucogingival Therapy defines mucogingival therapy as encompassing both surgical and non-surgical methods aimed at addressing issues with the form, placement, and volume of soft tissue and underlying bone. Furthermore, the report identifies periodontal plastic surgery as a surgical intervention designed to prevent or remedy defects and deformities in the gingiva, alveolar mucosa, or bone. These anomalies may arise from natural variations, developmental anomalies, trauma, or damage stemming from plaque-associated diseases (Table 1) [3,4].

**Table 1:** Classification of mucogingival deformities and conditions Modified from the American Academy of Periodontology 1999 Consensus Report NCCL: non-carious cervicel lesion

Mucogingival deformities and conditions around teeth
Periodontal biotype (thin scalloped, thick scalloped, or thick flat)
Gingival/soft tissue recession; facial or lingual surfaces, inter-proximal (papillary), severity of recession (Cairo recession type ((RT) 1, 2, or 3) gingival thickness, gingival width, presence of NCCL/cervical caries, patient esthetic concern (smile esthetic index), presence of hypersensitivity
Lack of keratinized gingiva
Decreased vestibular depth
Aberrant frenum/muscle position
Gingival excess; pseudo-pocket, inconsistent gingival margin, excessive gingival display, or gingival enlargement
Abnormal color

Connective tissue grafts (CTGs), initially developed as subepithelial connective tissue grafts (SECTGs) by Langer and Langer in 1985, are widely regarded as the most effective method in periodontal plastic surgery for achieving root coverage (RC) and enhancing tissue aesthetics [5,6]. However, several challenges related to CTGs, such as donor-site morbidity, limited tissue availability, and patient reluctance, have led to the exploration and development of allogeneic and xenogeneic alternatives [7].

One such option is the acellular dermal matrix (ADM), which is derived from processed human skin. ADM provides a scaffold that facilitates the colonization of host cells and promotes tissue incorporation without requiring palatal harvesting. Another alternative is the xenogeneic collagen matrix (XCM), sourced from animals like porcine or bovine tissue. This matrix undergoes processing to remove cells and reduce immunogenicity, offering a scaffold for tissue regeneration. Enamel matrix derivative (EMD), a protein extract from developing porcine teeth, has also been widely used. It stimulates the regeneration of periodontal tissue, including connective tissue and bone. Finally, platelet-rich fibrin (PRF) is a naturally derived material that releases growth factors to promote healing and tissue integration, offering a minimally invasive alternative to traditional grafts.

The above materials reduce donor-site morbidity, improve patient comfort, increase the availability of grafting materials, and enhance esthetic and functional outcomes in periodontal plastic surgery [8].

In 2009, Ehrenfest et al. [9] introduced a classification system for platelet concentrates depending on their fibrin and leukocyte content. This system includes (1) platelet-rich plasma (PRP), divided into pure PRP (P-PRP) and leukocyte- and platelet-rich plasma (L-PRP), and (2) PRF, further classified into pure PRF, leukocyte- and platelet-rich fibrin (L-PRF), and injectable PRF (I-PRF). PRF, introduced by Choukroun et al. in 2000, is a second-generation autologous platelet concentrate (APC) that is produced without the need for anticoagulants or additives [10]. Mourão et al. and Choukroun and Ghanaati introduced liquid I-PRF by adjusting centrifugation speeds and using non-glass tubes, which slows fibrin coagulation in the initial stages to create an injectable formulation [11,12]. Platelet concentrates are rich in growth factors and cytokines, promoting tissue regeneration, reducing inflammation, and enhancing wound healing [12-16].

Protocol for L-PRF, I-PRF, and advanced PRF (A-PRF)

**Figure 1:**
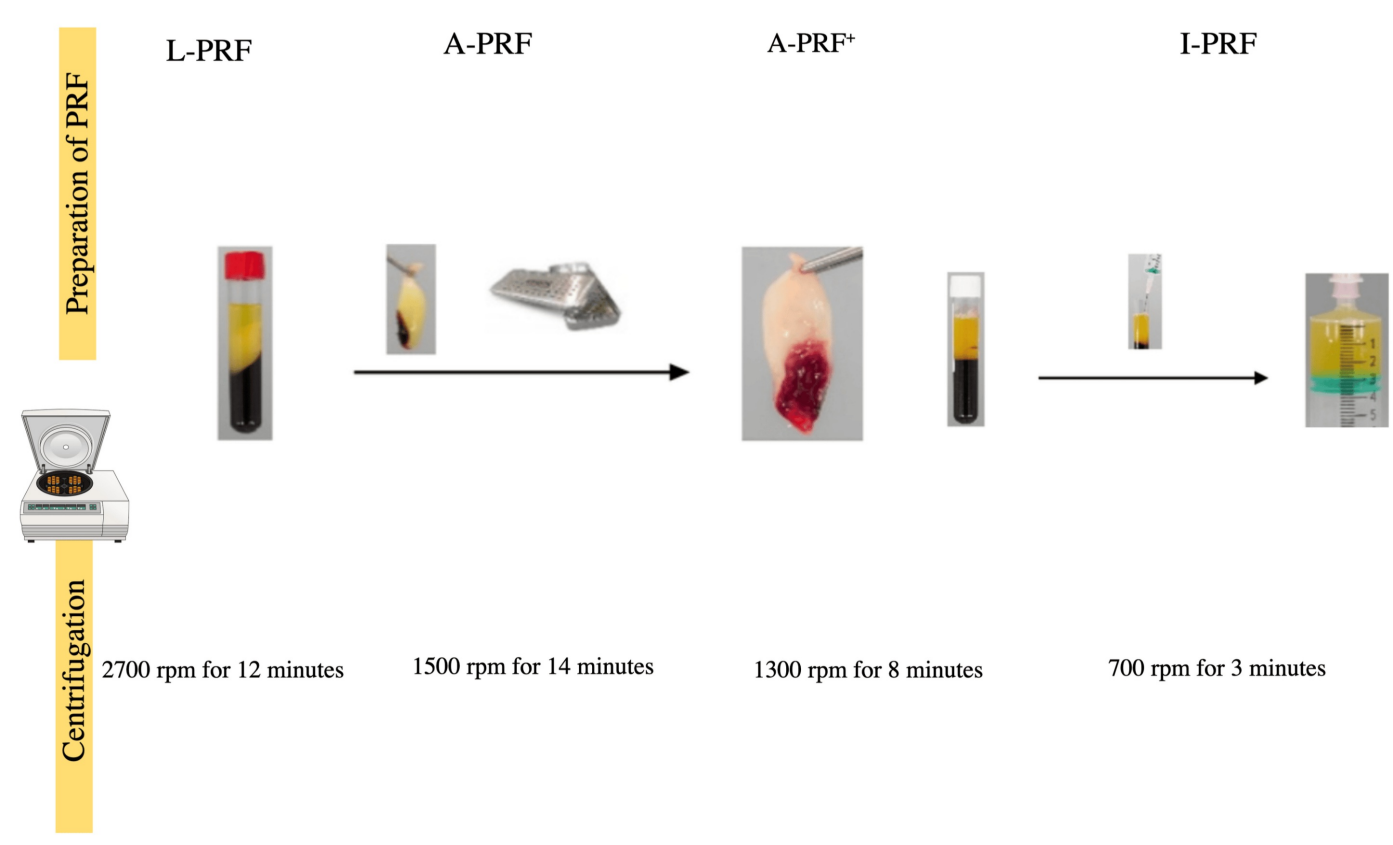
Types of PRF preparation PRF: platelet-rich fibrin, L-PRF: leukocyte- and platelet-rich fibrin, A-PRF: advanced platelet-rich fibrin, A-PRF+: similar to A-PRF but with reducing the speed and time which leads to PRF richer in growth factors, I-PRF: injectable platelet-rich fibrin, rpm: revolutions per minute Image Credit: Amal G. Jamjoom (created using Mind the Graph)

L-PRF, I-PRF, and A-PRF are being developed in regenerative medicine, particularly for dental and wound healing applications. Figure 1 shows the differences among these types of PRF.

A-PRF has a form similar to that of L-PRF; however, it is believed to have an enhanced biological profile due to its preparation method, which aims to preserve cellular components better. A-PRF releases growth factors at a rate that more closely mimics the natural healing process, potentially improving regenerative outcomes. A-PRF is used interchangeably with L-PRF, but due to its richer content of leukocytes and growth factors, it is believed to be superior for soft tissue healing [17].

The proper management and preparation of PRF are crucial for maximizing its healing capabilities. Using devices such as a PRF box can significantly improve the preservation of PRF, thereby enhancing its regenerative qualities for use in various healthcare settings. Creating PRF membranes using a PRF box begins by positioning the prepared fibrin clots inside the box. Next, the membrane tray can be placed over the clots. Applying pressure with the tray will compress the clots into flat fibrin membranes. Under physiological conditions, the primary release of active substances from PRF occurs several hours after preparation. Consequently, PRF remains effective for an extended period of time when stored under appropriate conditions [14,18].

## Review

The following literature review elucidates the substantial role of PRF and its derivatives, namely L-PRF, A-PRF, and I-PRF, in enhancing periodontal regeneration and healing. The review also highlights the diverse applications of PRF across various periodontal plastic surgery procedures, including RC, vestibuloplasty, enhancement of keratinized tissue width (KTW) around the teeth and implants, and gingival phenotype thickening.

Materials and methods

Eligibility Criteria

The included studies in the present review were selected based on the following criteria: peer-reviewed articles published in English, systematic reviews, studies on humans, and research specifically investigating the efficacy of PRF in periodontal plastic surgery procedures. The exclusion criteria were articles published in languages other than English, animal studies, and studies not directly related to PRF.

Search Strategy

A comprehensive search was conducted across multiple databases, including PubMed, Scopus, and Web of Science, from January 2019 up to June 2024. The search keywords included “platelet-rich fibrin," “periodontal regeneration," “root coverage," and “tissue healing.” The search strategy was adjusted for each database to utilize specific indexing terms and search features. The initial search yielded a total of 90 studies that appeared relevant based on their titles and abstracts. After screening the titles and abstracts for relevance, 22 studies were selected for full-text retrieval and further evaluation. These studies met the inclusion criteria, focusing on the efficacy of PRF in periodontal plastic surgery procedures. The remaining articles were excluded as they either did not meet the eligibility criteria, were included in the systematic reviews used in some parts of the review, or were not directly related to the subject matter. The final selection of 22 studies was included in the review, which provided insights into PRF's application in graft coverage, palatal donor site healing, RC, papilla reconstruction (PR), periodontal phenotype modification, and soft tissue preservation around dental implants. The follow-up period of the randomized clinical trials ranged from three months to a year.

Selection Process

The titles and abstracts of the selected articles were screened for relevance. Full texts of potentially relevant studies were retrieved and assessed against the inclusion criteria.

Data Collection Process

Data extracted from the included studies included author names, year of publication, study design, sample size, type of PRF used, and primary outcomes related to periodontal healing and regeneration.

Ethical Approval

Ethical approval was not required for the present study because neither human participants nor animals were included.

Applications

Graft Coverage

When applied to the graft site, the platelet concentrates accelerate the formation of new blood vessels and collagen, leading to faster and more predictable healing of the grafted tissue [17]. Keceli et al. found that incorporating PRF into the treatment involving coronally advanced flap (CAF) and CTG did not improve the overall results, except for a noticeable increase in tissue thickness [19].

Covering the Palatal Donor Site Following the Harvesting of a Free Gingival Graft (FGG)

There is conflicting evidence in the literature regarding the effect of PRF on palatal donor-site healing, bleeding, and postoperative pain after harvesting an epithelialized CT graft or a FGG. In their systematic review, Meza-Mauricio et al. suggested that PRF is beneficial in managing palatal wounds after harvesting FGGs, particularly in improving healing times, reducing pain, and controlling bleeding [20]. Nevertheless, the authors acknowledged that their findings should be approached carefully due to the restricted number of randomized trials and the diversity in the study methods [20]. In a 2019 randomized clinical trial by Sharma et al., it was determined that using a commercially available collagen dressing (CollaCote®) and a PRF membrane as a palatal bandage significantly enhanced wound healing for both tested groups. No notable differences were observed between the groups in terms of depth, hemorrhage, pain, epithelialization, and size of the wound. Nevertheless, initial healing was slightly better in the PRF group [21]. The results of another study by Kulkarni et al., in which PRF was compared to a regular periodontal pack for palatal coverage after FGG, showed complete wound closure in the PRF group within 14 days; furthermore, lower postoperative morbidity was reported in patients for whom PRF was used compared to patients for whom the periodontal pack was used [22]. Mutallibli et al. determined that neither L-PRF nor A-PRF yielded any significant results compared with periodontal pack + palatal stent [23]. Scott et al. [24]. reported a lack of differences in early palatal wound healing, patient pain perception, or analgesic consumption between the use of PRF and a hemostatic agent (ActCel material, Coreva Health Science, North Rhine-Westphalia, Germany) as donor-site wound dressings. De Almeida et al. also concluded that all treatment modalities for palatal donor-site coverage (low-level laser therapy, cyanoacrylate tissue adhesives, and ozone therapy) improved healing [25].

RC

Flap design (coronally advanced): A significant number of systematic reviews comparing PRF to CTG or other substitutes have been published: Mancini et al. (comparing the efficacy of L-PRF in addition to CAF for the treatment of both single and multiple gingival recessions (GRs) compared to the CAF alone and to the adjunct of CTG), Moraschini et al. (comparing CAF alone to ADM, XCM, EMD, and PRF), Castro et al. (comparing L-PRF to CTG), Li et al. (comparing different types of the APCs combined with CAF to CAF alone), Rodas et al. (comparing CTG to PRF), and Panda et al. (comparing CAF alone to CAF + PRF) have published systematic reviews on PRF efficacy for GRs treatment [26-31].

The primary outcomes evaluated in these evaluations were the average RC (mRC), reduction in recession, gain in KTW, increase in gingival thickness (GT), and patient-reported outcome measures, like pain perception and discomfort. However, the aforementioned reviews are subject to several limitations. Firstly, the number of included studies was relatively small, which may limit the generalizability of the findings. Additionally, some of the included studies presented a high risk of bias, further complicating the interpretation of the results. Another limitation is that the analysis often combined data from both single-site and multiple-site studies, which could introduce variability in the outcomes. Moreover, various types of platelet concentrates, including PRP and concentrated growth factors, were used in conjunction with PRF, increasing the complexity. Lastly, some reviews focused solely on the effects of L-PRF in comparison to CAF procedures without making direct comparisons to CTG, which is widely regarded as the most effective technique in RC procedures [31].

Based on the findings of the previous reviews, CTG provides the most favorable clinical and esthetic outcomes (overall higher mRC, KTW gain, and GT gain for CAF + CTG); however, these findings did not reach statistical significance in some of the included studies.

The authors of more recent studies, such as a study conducted by Yavuz et al. [32], have concluded that L-PRF + CAF can be considered a good alternative to CTG + CAF for RC when increased KTW or GT is not required.

Moraschini et al. conducted a systematic study that examined several alternatives for CTG. They found that utilizing a combination of biomaterials improves the efficiency of RC compared to using only CAF. According to the ranking of treatments, all the tested biomaterials had a good impact on RC; however, ADM was determined to have the most favorable outcomes [33]. Miron et al. systematically reviewed PRF efficacy in multiple regenerative procedures, including RC. They concluded that PRF usage for the treatment of GR is limited; however, they noted that follow-up studies are required owing to the beneficial effect of PRF on tissue healing [34]. Amine et al. conducted a systematic review to compare different substitutes (ADM, XCM, EMD, and PRF) to CTG for Miller class I and II GR treatment. They concluded that combining the above materials with CAF might be considered an alternative to CTG but with a high level of uncertainty [35]. Jankovic et al. discovered that PRF utilization leads to lower morbidity and faster wound healing than EMD, but EMD leads to better keratinized KTW [36].

Flap design (vestibular incision subperiosteal tunnel access (VISTA)): Boddu et al. showed in their study that using PRF in VISTSA resulted in better GT than using VISTA alone [37]; in comparison, Al Kababji et al. showed that PRF did not enhance the effect of VISTA in the treatment of multiple GRs [38].

PR

PRF vs. CTG: In their studies, Barakat et al. and Singh et al. stated that PRF can be considered a viable alternative to CTG in PR [39,40]. In another study by Gadi et al., the efficacy of the micro-tunneling technique using either CTG or PRF for PR was evaluated. The study concluded that using CTG and PRF in the micro-tunneling technique is comparably effective for reconstructing the interdental papilla and reducing black triangles without significant differences in the outcomes between the two biomatrices [41]. On the other hand, a study by Ozcan Bulut et al. evaluated the effectiveness of PRF and CTG in PR using the semilunar incision technique in the maxillary anterior region. The results indicated that while both PRF and CTG treatments led to improvements in most periodontal parameters, CTG was found to be more effective in maintaining KTW. Overall, CTG demonstrated better outcomes in PR with less risk of recurrence, leading to the conclusion that CTG is preferable over PRF for this specific treatment [42]. Another study by Sharma et al. comparing CTG and PRF using the Han and Takie technique [43] for PR showed better results with CTG than PRF regarding enhancing papilla dimensions [44].

PRF with micro-needling (MN): L-PRF and I-PRF benefit tissue regeneration and are less invasive, rendering them suitable for increasing the GT and width of keratinized tissue in thin gingival biotypes using micro-needles.

MN, sometimes called collagen induction therapy, is a treatment modality that involves using extremely thin, short, and sterile needles to puncture the skin. Such needles have a typical diameter of 0.5-2.5 mm. The fundamental concept of MN involves the deliberate application of physical trauma to the skin. This method utilizes tiny needles that penetrate the skin, creating micro-injuries that initiate a healing response in the dermis. As the needles puncture the stratum corneum, they form small channels, referred to as micro-conduits, while causing minimal damage to the epidermis. This action stimulates the release of growth factors that enhance the synthesis of collagen and elastin in the papillary layer of the dermis. The natural wound healing mechanisms are then activated, attracting platelets and neutrophils that release growth factors such as TGF-alpha, TGF-beta, and platelet-derived growth factors, leading to the deposition of new collagen by fibroblasts. Utilization of the above treatment can help minimize the visibility of scars and hyperpigmentation, reduce fine lines and wrinkles, tighten loose skin, improve skin texture and overall complexion, and diminish the appearance of large pores.

A randomized clinical trial conducted by Zaaya et al. provides valuable insights into the efficacy of MN vs. ADM in treating RT1 GR in patients with thin gingival phenotypes. The study authors concluded that MN and a CAF could be a practical, graftless approach for increasing GT and KTW for treating RT1 GR defects. This method is comparable to the traditional use of ADM with CAF, providing a less-invasive alternative with similar efficacy. More in-depth research is recommended to refine clinical MN protocols and explore the long-term outcomes of the technique compared to those of other grafting materials [45].

Periodontal Phenotype Modification

In a study by Veluri et al., researchers assessed the effectiveness of combining I-PRF with MN vs. FGG in modifying the periodontal phenotype. The findings revealed that I-PRF and MN together deliver clinical outcomes equivalent to FGG in transforming the periodontal phenotype. The study also highlighted that this method is less invasive, has lower morbidity, and provides better aesthetic outcomes compared to FGG [46]. In their study comparing I-PRF to no treatment for thin phenotype modification after three months of follow-up, Manasa et al. concluded that I-PRF is a valuable treatment for phenotype thickening [47]. However, their results should be interpreted carefully due to the short-term follow-up.

Laser Depigmentation

A randomized controlled split-mouth study by Dahiya et al. compared Choukron’s L-PRF to non-eugenol periodontal dressing, made of zinc oxide or other inert materials that don’t cause tissue irritation, for gingival depigmented site coverage. The study's results indicated that PRF treatment resulted in significantly better wound healing outcomes, as evidenced by lower pain scores, higher healing index scores, and better epithelization than those reported for non-eugenol periodontal dressing. Moreover, the results of histological analyses showed that the PRF-treated sites had less inflammatory cell infiltration, thus indicating superior healing potential [48].

The results of another randomized controlled split-mouth clinical trial conducted by Ibrahim et al. discovered that the laser gingival depigmentation followed by local injection of I-PRF enhanced normal healing processes, as evidenced by histological analyses within the first week. This treatment also reduced patient discomfort and pain and positively affected patient satisfaction with the utilized technique. While the pigmentation score did not significantly change during the first three months, the overall healing and patient experiences were better with the I-PRF treatment than with the control sites that received no treatment [49]. Finally, patients who underwent PRF treatment showed comparable healing and re-epithelialization results to those treated with hyaluronic acid after gingival depigmentation [50].

Soft Tissue Preservation Around Dental Implants

In their study, Azangookhiavi et al. found significantly less GR around dental implants compared with freeze-dried bone allografts after the ridge preservation procedure and implant placement [51]. Another randomized controlled study by Cheruvu et al. involved 40 patients who received non-submerged dental implants with or without PRF and suggested that PRF membranes could be beneficial in improving the outcomes of dental implant procedures by promoting better soft tissue healing and potentially reducing crestal bone resorption [52]. Based on a study done by Boora et al., PRF demonstrates a synergistic effect on peri-implant bone formation and tissue healing in the maxillary anterior region when used in single-stage implant placement with immediate provisionalization [53].

## Conclusions

The use of PRF in periodontal plastic surgery offers several therapeutic advantages, such as a notable acceleration of wound healing, reduction in postoperative pain, and better management of bleeding, particularly at palatal donor sites, following FGG harvesting and gingival depigmentation procedures. These benefits are primarily attributed to the rich content of growth factors, cytokines, and leukocytes in PRF, which collectively provide a conducive environment for tissue regeneration.

Moreover, PRF has shown promising outcomes in improving GT and attachment levels when used with CAF procedures. Although some researchers have noted variability in outcomes such as keratinized mucosal width, the overall evidence supports the effectiveness of PRF for use in periodontal therapy, especially when combined with surgical interventions. However, the studies reviewed herein should be interpreted with caution due to differences in PRF centrifugation and application protocols.
